# Additive Manufacturing to Mimic the Nonlinear Mechanical Behavior of Cardiac Soft Tissue

**DOI:** 10.3390/polym17212949

**Published:** 2025-11-05

**Authors:** Sara Valvez, M. Oliveira-Santos, L. Gonçalves, A. P. Piedade, A. M. Amaro

**Affiliations:** 1 University of Coimbra, Centre for Mechanical Engineering, Materials and Processes (CEMMPRE, ARISE), Department of Mechanical Engineering, 3030-788 Coimbra, Portugal; sara.valvez@dem.uc.pt (S.V.); ana.piedade@dem.uc.pt (A.P.P.); 2 University of Coimbra, Institute for Clinical and Biomedical Research (iCBR), Faculty of Medicine, 3000-548 Coimbra, Portugal; manuel_ol_santos@hotmail.com (M.O.-S.); lgoncalv@ci.uc.pt (L.G.)

**Keywords:** additive manufacturing, nonlinear material behavior, soft tissue mechanics, parametric optimization, left atrial appendage

## Abstract

Soft biological tissues display highly nonlinear and anisotropic mechanical behavior, which is critical to their physiological function. Replicating these mechanical properties using engineered materials and additive manufacturing represents a significant challenge in biomedical engineering, particularly for surgical simulation, device development, and preclinical testing. The left atrial appendage (LAA) was selected since it plays a central role in thrombus formation during atrial fibrillation, significantly contributing to cardioembolic stroke. This study proposes a framework for reproducing the nonlinear stress–strain behavior of soft tissue using 3D-printed models. The methodology integrates experimental material selection with optimization of key printing parameters to ensure structural reliability and functional mechanical performance. Two polymers—polyurethane (TPU) and a thermoplastic with elastomer-type behavior (TPE)—were selected for their tunable hardness and elasticity. A parametric study was conducted to investigate the effects of Shore A hardness (60A to 100A), infill density (0% to 100%), and external shell number (zero to two) on the tensile performance of printed models. Mechanical testing was performed to extract stress–strain curves and evaluate the mechanical response. The practical implications of this study are significant, demonstrating the potential of additive manufacturing for anatomical reproduction and replicating functional mechanical properties in soft tissue models.

## 1. Introduction

**Figure 1:**
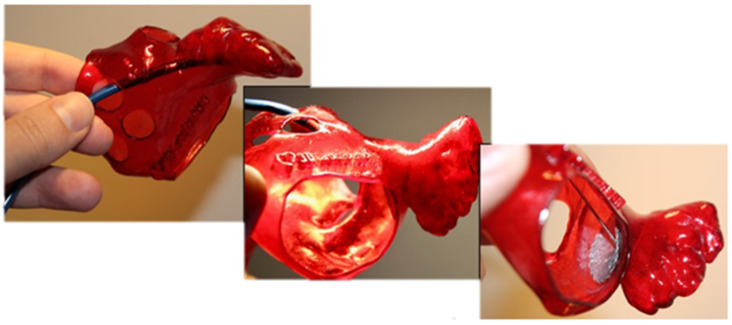
LAA occlusion planning: appropriate device sizing is supported by a synthetic model.

Soft biological tissues are fundamental to the structural and functional integrity of physiological systems, particularly within the cardiovascular domain, where they are subjected to dynamic loading and large deformations. These tissues exhibit complex mechanical behavior characterized by nonlinearity, anisotropy, and viscoelasticity, typically represented by a J-shaped stress–strain response [1]. Accurately reproducing such behavior in synthetic analogs remains a significant challenge in engineering concepts, especially in developing models intended for surgical simulation, device testing, and computational analysis [2,3,4]. A clinically relevant example of such tissue is the left atrial appendage (LAA), a compliant cardiac structure recognized as the primary site of thrombus formation in patients with atrial fibrillation (AF) and thus a major contributor to cardioembolic stroke [5]. Owing to its critical role in thromboembolic risk, the LAA has become a principal target for deploying occlusion devices, which aim to mechanically isolate the appendage from systemic circulation and thereby reduce stroke incidence. Figure 1 illustrates the role of a synthetic LAA model in supporting occlusion planning, where the replication of patient-specific anatomy enables accurate device sizing and fit assessment.

Although LAA models have already been integrated into interventional cardiac simulators, the literature reveals two predominant approaches. The first focuses primarily on anatomical reliability, aiming to reproduce the geometry of the appendage for visual and spatial assessment of device fit [6]. These models are typically made from rigid materials and neglect the mechanical behavior of native tissue. The second approach explores the use of flexible materials to mimic the tissue’s mechanical properties [7]. However, inadequate replication of the tissue’s stress–strain behavior may lead to unrealistic device–tissue interactions during simulation, resulting in incorrect estimation of device type, size, or positioning. If the mechanical properties and stress–strain behavior of the model do not accurately replicate those of the native tissue, device–tissue interaction during simulation may be unrealistic, potentially leading to incorrect assessment of device fit and anchoring. Consequently, it may compromise the effectiveness of training or planning and misrepresent potential clinical risks such as peri-device leakage, device embolization, incomplete closure, or excessive stress on the atrial wall [8]. These limitations underscore the need for a comprehensive mechanical characterization, particularly in terms of reproducing the nonlinear stress–strain response essential for simulating realistic device–tissue interactions.

Cardiac tissues, including the LAA, exhibit complex mechanical behavior characterized by anisotropy, nonlinear elasticity, and viscoelasticity [8]. Specifically, the LAA displays high strain capacity and distensibility, which are essential for its role as a decompression chamber and for modulating atrial function [9]. Strain capacity reflects tissue contractility and reduced strain is associated with impaired function and increased thrombus risk in atrial fibrillation [10]. Distensibility, the ability to accommodate volume under pressure, is linked to tissue elasticity and prevents blood stasis under hemodynamic load [11]. These functional demands translate into engineering requirements for materials capable of large, reversible deformations with minimal plasticity. In engineering terms, this behavior is characterized by a material’s ability to undergo large reversible deformations within the elastic domain. Therefore, materials selected to mimic the LAA should exhibit high elasticity and preserve mechanical integrity at large strains. These requirements exclude materials with limited deformability, or predominant plastic behavior. Accordingly, elastomeric materials are the most suitable candidates for mimicking the mechanics of LAA tissue. Their inherent flexibility, ability to recover after deformation, and tunable Shore hardness make them ideal for reproducing the nonlinear, highly elastic response of native cardiac tissue [12]. The literature on this subject mentions the use of polymeric materials and additive manufacturing (AM) technologies to create customized, patient-specific models of the LAA [7,13]. Among the published papers, the use of flexible materials to produce these models was associated with better procedural outcomes [13]. Materials with rubber-like properties, such as Tango^®^ (from Stratasys Ltd., Eden Prairie, MN, USA) [6,14,15,16], polyurethane (TPU) in filament form, gelatin [17], silicone [18,19] and Latex [20] in the liquid state, are reported in the literature. Although flexible materials have shown improved realism in procedural outcomes, a significant number of these studies lack specification of the materials used or fail to provide systematic mechanical characterization [21,22,23,24]. In other works, one from the authors, it was found that thermoplastic polyurethane (TPU) exhibited mechanical properties closer to those of LAA tissue than other tested materials [12,25]. However, deviations in the stress–strain response, particularly in curve shape and modulus, highlighted the need for further refinement.

**Figure 2:**
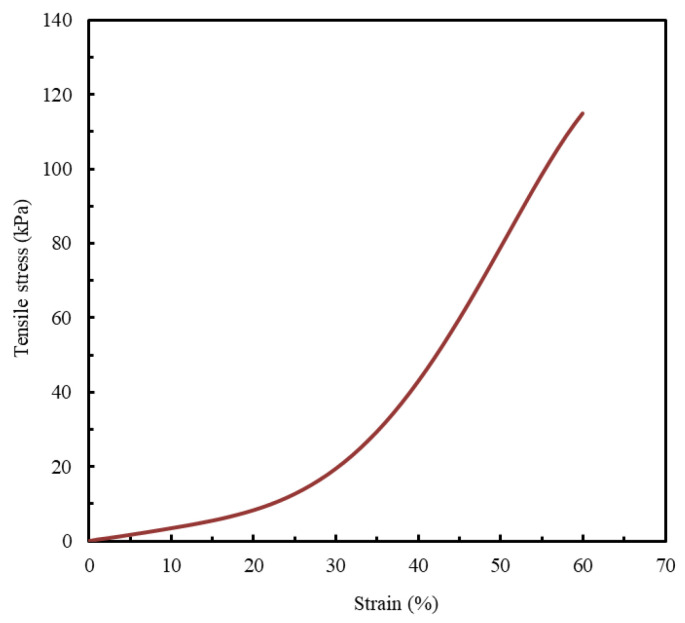
LAA occlusion planning: appropriate device sizing is supported by a synthetic model. Tensile stress- strain curve. Adapted from [1].

Since quantitative data on the mechanical behavior of biological LAA tissue are not widely available in the published literature, this study adopts the only experimentally obtained stress–strain curve identified, to the best of the authors’ knowledge, as a reference benchmark for model evaluation (Figure 2) [1].

In addition to material selection, the manufacturing process plays a decisive role in determining final mechanical and surface characteristics. Given the LAA’s anatomically complex and highly variable morphology, conventional manufacturing methods fall short in replicating its geometry and mechanical functionality. In this context, AM offers significant advantages, including reproducing complex anatomical geometries and fine-tuning mechanical properties through precise control of processing parameters. While stereolithography (SLA) offers smooth, high-resolution parts, fused filament fabrication (FFF) allows greater material flexibility and produces textured surfaces more aligned with the morphology of cardiac tissues [26]. For these reasons, FFF was selected in this study to fabricate tissue-mimicking specimens.

Although AM has advanced in biomedical applications, systematic efforts to optimize material-process combinations for replicating the nonlinear tensile behavior of cardiac tissues, particularly the LAA, remain limited, especially regarding mechanical properties.

This study aims to fill that gap by developing a framework capable of replicating the nonlinear mechanical behavior of LAA biological tissue. A parametric approach is employed to investigate the influence of key parameters that significantly affect structural integrity, namely Shore A hardness, infill density, and shell number, on the tensile response of FFF-printed elastomeric specimens. The goal is to identify optimized material–structure combinations suitable for future implementation in anatomically accurate LAA models, enhancing the realism of mechanical behaviors and the functional relevance of cardiovascular simulators and supporting more effective, patient-specific device evaluation.

## 2. Materials and Methods

### 2.1. Materials

Thermoplastic polymers with elastomeric-like behavior (TPU and TPE) were selected based on their Shore hardness, specifically TPU 60D, TPU 93A, TPU 40D, TPU 85A, TPE 70A, and TPE 60A. To enable a direct comparative analysis, hardness values in Shore D values were converted to Shore A, as this scale is more appropriate for measuring the hardness values of softer materials. Therefore, throughout this study, TPU 60D and TPU 40D will henceforth be referred to as TPU 100A and TPU 90A, respectively. The polymers were purchased from Filament2Print (Pontevedra, Spain) in filament form, with a diameter of 1.75 ± 0.04 mm.

### 2.2. Material Selection and Optimization of Printing Parameters

**Figure 3:**
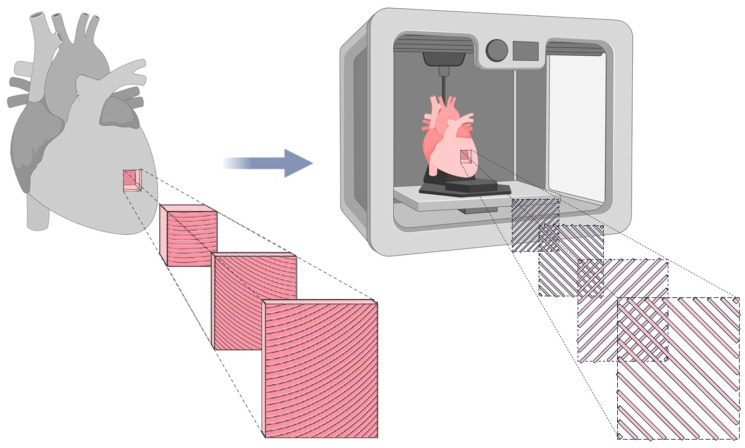
Representation of cardiac tissue anisotropy and its biomimetic counterpart fabricated using FFF. The biomimetic model utilizes a 45°/−45° infill pattern to replicate the structural alignment of natural cardiac tissue. Adapted from [29].

Since the primary objective of this study is to replicate the mechanical behavior of biological LAA tissue, it is essential to reproduce its anisotropic nature, a fundamental characteristic of cardiac tissue [27]. Cardiac muscle fibers are arranged in helical and oblique layers, which result in multidirectional load paths not limited to the Cartesian 0° and 90° axes [28]. Consequently, to mimic this structure, a 45°/−45° infill orientation was applied, allowing a more realistic simulation of fiber alignment and mechanical behavior, as illustrated in Figure 3.

Although the 45°/−45° pattern does not replicate the full helicoidal complexity of the cardiac tissue, it offers mechanical advantages over 0°/90° configurations by improving stress distribution, reducing localized failure, and enhancing fatigue resistance. This infill strategy provides a more uniform mechanical response, better mimicking the anisotropic nature of the LAA under physiological loads [30].

Healthy cardiac tissue is typically orthotropic, with stiffness varying in three orthogonal directions based on the alignment of fibers, laminar sheets, and the extracellular matrix [31]. In contrast, the LAA in patients with atrial fibrillation often exhibits anisotropic behavior due to fibrotic remodeling, resulting in directional mechanical heterogeneity [32]. Therefore, the aim is not to reproduce an ideal orthotropic model, but rather a controlled anisotropic response aligned with pathological LAA tissue mechanics.

The 45°/−45° filament orientation introduces directional reinforcement, simulating the tensile, compressive, and torsional stresses encountered in vivo. This pattern reduces stress concentration and improves resistance to multi-axial loads, which is especially relevant for models subjected to cyclic loading conditions. Compared to 0°/90°, it offers a mechanical compromise across multiple directions, approximating the elastic and fatigue behavior of native LAA tissue [33,34]. Thus, the selected infill strategy supports the development of more mechanically faithful models for use in simulation-based planning of LAA occlusion procedures.

In the first stage of this study, the material selection process, the specimens were fabricated using an original Prusa MK4S (Prusa Research, Prague, Czech Republic) and fixed printing parameters, including a layer height of 0.2 mm, two shells, 100% infill density, and an infill pattern of lines oriented at +45°/−45°. The remaining parameters varied according to the values presented in Table 1 and the supplier’s recommendations.

**Figure 4:**
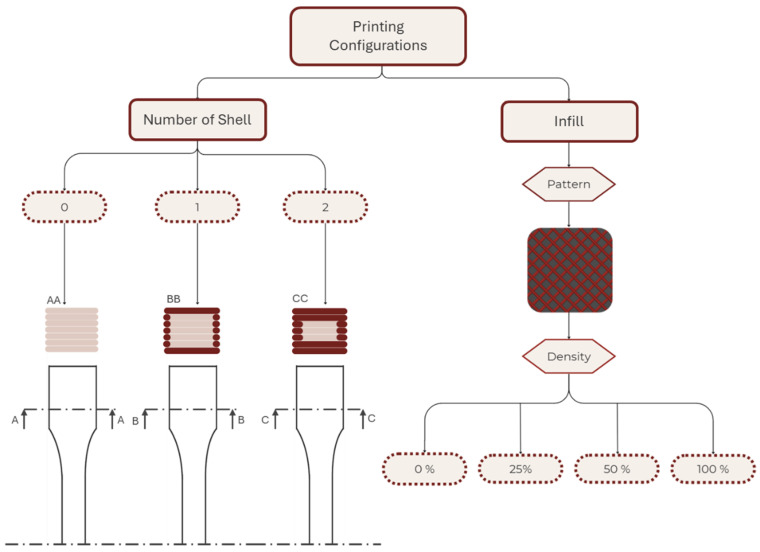
Schematic representation of the printing parameters used in the second part of the study.

The second stage of the research aimed to evaluate the impact of the two most critical printing parameters on the structural integrity of AM models: the number of shells and the infill density [35,36]. Figure 4 schematizes the combination of the printing parameters used in addition to the fixed parameters of the first part.

Variations in the printing parameters were intended to assess the impacts of the shell number (zero, one, and two) and infill density (D) (0%, 25%, 50%, and 100%) on the mechanical properties of specimens. Shell number is defined as equal to the perimeter contours (Np), and in this study, the numbers of top solid layers (T) and bottom solid layers (B) were set to be equal to the shell number (i.e., shell number = Np = T = B). D was specified independently. Specimens were fabricated with 0, 1, or 2 shells; accordingly: 0 shell (infill-only: Np = 0, T = 0, B = 0, D > 0), 1 shell (Np = 1, T = 1, B = 1), and 2 shells (Np = 2, T = 2, B = 2). Multiple samples were fabricated for each selected material and set of printing parameters to ensure at least five valid test results for each material–printing-parameter combination.

### 2.3. Mechanical Characterization

When studying mechanical properties for designing cardiovascular medical simulators, it is essential to emphasize the role of radial resistance and the material’s ability to anchor an occlusion device. Radial resistance is described as the capacity of a material to withstand deformation when subjected to compressive loads from multiple directions simultaneously [37]. It is considered a critical characteristic for accurately mimicking the function of the LAA, since this biological structure undergoes significant deformations and sustains pressures during the cardiac cycle [9]. Therefore, radial resistance emerges as a critical mechanical property in the planning and production of LAA models. The existing literature suggests that tensile tests provide a means to evaluate radial strength and stiffness, with established direct correlations between these parameters and other material properties, such as ultimate stress (σ) and Young’s modulus (E), respectively [38]. Additionally, since the results used as a control for the current study were derived from a tensile stress–strain curve (Figure 2) [1], tensile tests were selected as the most appropriate characterization technique.

**Figure 5:**
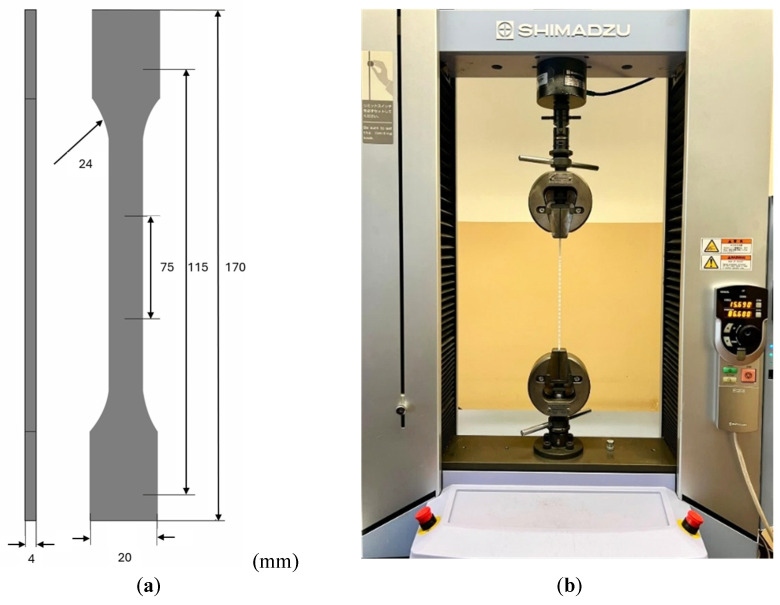
(**a**) Schematic representation of the samples according to ISO 527 standard [39]. (**b**) Experimental tensile test setup using Shimadzu Autograph AGS-X.

The specimens were produced and characterized using tensile tests in accordance with the ISO 527 standard [39]. Figure 5a illustrates the geometry used for the samples in the experiments. For each material, at least five specimens were produced, following the configuration shown in Figure 5a, to ensure repeatability and reproducibility in the analyses performed. Figure 5b depicts the experimental setup, which utilized Shimadzu Autograph AGS-X universal tensile test equipment equipped with a 5 kN load cell and associated with TrapeziumX software (Version 1.5.1). The tests were conducted at a grip speed of 5 mm/min under room temperature conditions.

The stress–strain curves obtained from the tensile tests were analyzed to determine the critical mechanical properties for replicating LAA tissue’s mechanical behavior, including the maximum tensile stress (σ), Young’s modulus (E), and maximum strain (ε). The study will compare the stress–strain behavior of the printed specimens to the documented mechanical behavior of LAA biological tissue reported in the literature to validate the suitability of the results (Figure 2).

## 3. Results and Discussion

This section, divided into three subsections, presents and discusses the results regarding material selection, the influence of the number of shells and infill density, and the replication of the mechanical behavior of the LAA biological tissue.

### 3.1. Material Selection

**Figure 6:**
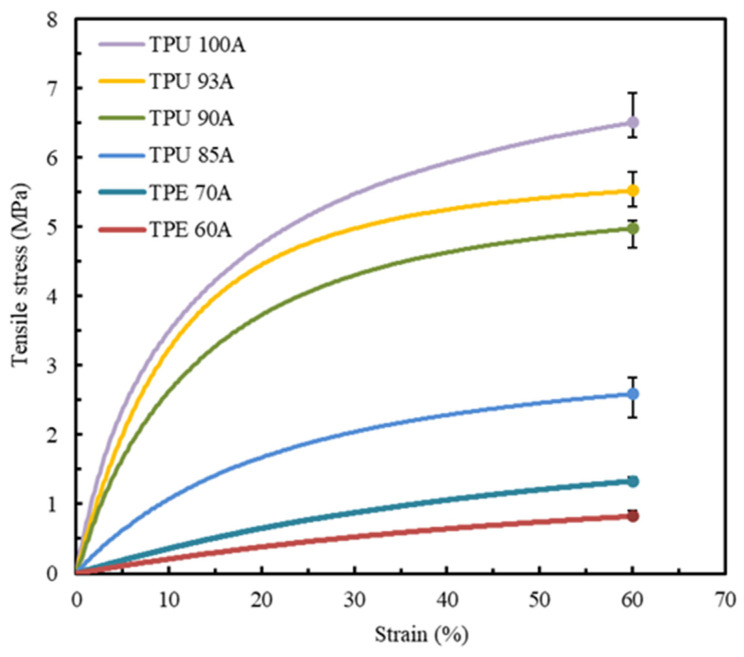
Average stress–strain tensile curves of all the synthetic polymers studied, with two shells and 100% infill.

The first experimental tests were conducted to determine the tensile properties of the six polymers referred to before: TPU 100A, TPU 93A, TPU 90A, TPU 85A, TPE 70A, and TPE 60A. At least five valid tests were conducted for each combination of material and printing parameters, with the resulting strain-stress curves demonstrating a high level of repeatability. Figure 6 presents the average stress–strain curves for each material, with each sample produced using two shells and 100% infill density.

Table 2 displays the average and standard deviation values of the tensile properties of the six pre-selected materials, particularly the maximum tensile stress (σ) and Young’s modulus (E).

A strain limit of 60% was established as the comparative benchmark for all analyzed materials. This value is aligned with a study that reported that the stress–strain curve of the LAA terminates at a similar strain value [1].

The low variability of the results is consistent across all the materials analyzed, as shown by the low dispersion represented in Figure 6 and Table 2. The stress–strain curves of the materials exhibit different mechanical behaviors, with all materials demonstrating higher stress at a strain of 60%. TPU materials show an ascending convex curve, indicating a significant increase in tensile stress with increasing strain. Additionally, it is possible to observe that all the TPU materials reveal a higher strain resistance when compared to TPE. TPU 100A, TPU 93A, and TPU 90A exhibit a steeper stress–strain curve with a maximum stress of around 6.8 MPa, 5.6 MPa, and 5 MPa, respectively. On the other hand, TPU 85A, TPE 70A, and TPE 60A exhibit flatter curves, which show lower tensile stress and stiffness and, consequently, higher deformation capacities. While TPU 85A can achieve a maximum tensile stress value of approximately 2.7 MPa, TPE 70A only reaches 1.3 MPa, a difference of around 52%. Among all the tested materials, TPE 60A shows the lowest stress of about 1 MPa and, according to its stress–strain curve evolution, the lowest Young’s modulus. The results indicate that none of the combinations of material-processing parameters allow for an accurate and direct replication of the mechanical behavior of LAA biological tissue. Consequently, aiming to attain the tensile values and the geometry of the stress–strain curve of the control specimen, only TPU 85A, TPE 70A, and TPE 60A were selected for further study.

### 3.2. Influence of the Number of Shells and Infill Density

**Figure 7:**
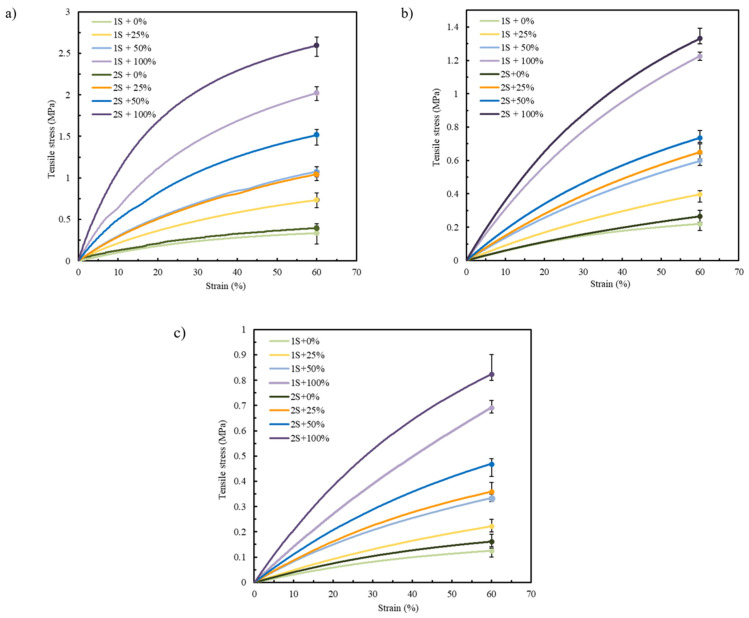
Average stress–strain tensile curves for (**a**) TPU 85A, (**b**) TPE 70A, and (**c**) TPE 60A.

Focusing on the pre-selected materials TPU 85A, TPE 70A, and TPE 60A according to the results presented in Section 3.1, only two printing parameters were varied, the number of shells and infill density, with the aim of mimicking the mechanical behavior of biological tissues [12,40]. Figure 7a–c display the tensile stress–strain curves of TPU 85A, TPE 70A, and TPE 60A, respectively. “1S” and “2S” refer to the number of shells (one and two, respectively), while the percentages (0%, 25%, 50%, and 100%) indicate the infill density.

Table 3 displays the stress (σ) and Young’s modulus (E) of TPU 85A, TPE 70A, and TPE 60A considering the previously mentioned variations in printing parameters, specifically in terms of shells and infill density.

According to the results in Figure 7 and Table 3, the mechanical behavior of each printed sample was strongly influenced by the selected printing parameters, regardless of the material. This highlights the critical role of these parameters in determining the structural integrity of the printed specimens. The literature consistently reports that shell number and infill density are among the most influential parameters affecting the mechanical performance of FFF-printed parts [41]. Specifically, increasing the number of outer shells leads to higher tensile strength, as the shell region functions as the primary load-bearing structure [42,43].

#### 3.2.1. Shell Number

A greater shell number results in denser and mechanically more stable specimens, improving their capacity to withstand tensile loading [36]. However, there appears to be a saturation point beyond which further increases in shell number do not translate into significant gains in strength, potentially due to manufacturing-related defects or internal stress concentrations introduced during the printing process [44]. Moreover, shell continuity is essential: interrupted or poorly bonded shells can significantly reduce a specimen’s ability to carry loads, even when other parameters are favorable [45].

Within the range of shells tested (one and two), the findings of the current study corroborate previous studies [35,44,46], confirming that a greater shell number resulted in a clear and consistent impact on the tensile behavior of the specimens. This reinforces its role as a critical parameter in optimizing the mechanical performance of structures produced via FFF [35,36].

In Figure 7, specimens printed with 2S consistently exhibit higher tensile strength compared to those with 1S. This tendency can be attributed to the increased structural stiffness provided by the additional shell material. Given that the shells are oriented longitudinally to the direction of tensile loading, they offer enhanced resistance, allowing for more effective load transfer along the length of the specimen. This behavior reflects the intrinsic advantage of filament alignment in the load direction, which promotes higher strength and stiffness under uniaxial stress [47].

In the case of TPU 85A, the difference between 1S and 2S becomes more pronounced as strain values increase, with 2S exhibiting a steeper curve, reaching tensile stress values higher than 2.6 MPa in samples with 100% infill density. A similar tendency is observed in the specimens printed using TPE 70A and TPE 60A, although they have lower tensile stress values due to their more ductile behavior. TPE 70A and TPE 60A samples with 2S and 100% infill show tensile stress values of approximately 1.33 MPa and 0.75 MPa, respectively. These values contrast with those with only 1S, which exhibit tensile stresses around 1.26 MPa and 0.5 MPa, respectively, highlighting their reduced ability to withstand higher stress values, which means a decrease of about 5.3% and 33.3% when comparing the 2S and 1S specimens for TPE 70A and TPE 60A, respectively.

#### 3.2.2. Infill Density

The second parameter evaluated, infill density, also significantly affects the mechanical response of FFF-printed parts [48]. Higher infill densities generally increase tensile strength and stiffness, while lower densities promote more flexible behavior, which the results support. TPU 85A specimens showed the highest tensile strength at greater infill percentages in this study, due to improved internal load distribution [49]. The same trend was observed in TPE 70A and TPE 60A, although their lower hardness resulted in lower stress values overall.

As shown in Figure 7, while all tested materials tolerated large deformations (up to 60% strain), neither the stress–strain curves nor the maximum stress values fully replicated the nonlinear mechanical behavior of native LAA tissue. Accordingly, further optimization is required to achieve accurate mechanical biomimicry.

### 3.3. LAA Tissue Mechanical Behavior Replication

In agreement with the literature and as confirmed by the results presented in Figure 7, the number of shells has a pronounced influence on structural integrity and stress–strain curve geometry [50,51]. However, the mechanical behavior of LAA biological tissue was not achieved with either of the selected combinations of printing parameters. Considering that both one and two shells offered greater structural integrity to the samples, the decision was made to exclude shells in subsequent analyses. Therefore, the next phase of this study will be conducted using zero shells to further investigate the material’s mechanical properties without the influence of outer layers, focusing on examining the impact of infill density.

Building upon preliminary tests conducted with 0%, 25%, 50%, and 100% infill, a 25% density emerged as a promising compromise between mechanical performance and material efficiency, particularly when using zero shells. However, to further explore the lower limits of structural integrity while preserving printability, a 10% infill density was additionally introduced. As a result, two configurations were selected for analysis: zero shells with 10% infill (0S + 10%) and zero shells with 25% infill (0S + 25%). These tests aimed to evaluate the sensitivity of the material’s mechanical response to internal structure variations under minimal reinforcement conditions.

**Figure 8:**
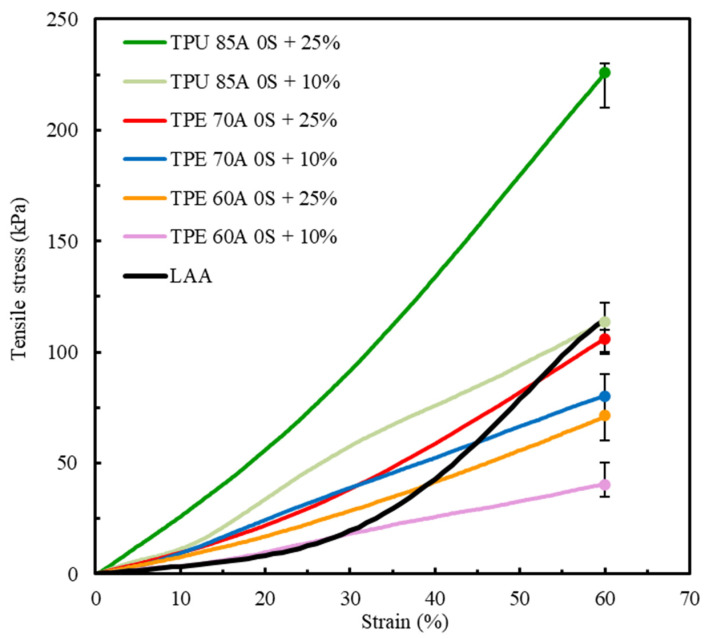
Comparison of the average stress–strain tensile curves between TPU 85A, TPE 70A and TPE 60A (this study) and LAA biological tissue [1].

Figure 8 shows a comparative analysis of the mechanical behavior of TPU 85A, TPE 70A, and TPE 60A with different infill density percentages (10% and 25%) and biological LAA tissue, which was considered the reference for this study [1].

Table 4 displays the maximum σ and E values of TPU 85A, TPE 70A, and TPE 60A for samples with the printing parameters 0 S + 10% and 0% + 25%.

The mechanical properties of cardiac biological tissues vary significantly depending on the tissue type (e.g., myocardium, cardiac valves, pericardium, etc.) and the experimental conditions (e.g., physiological state, stretch direction, etc.) [52,53,54]. Despite these varying conditions, the mechanical properties of these tissue types predominantly fall within the kPa range, maintaining consistency in their order of magnitude [55].

Matching the mechanical behavior of the synthetic LAA model to that of the biological tissue is essential and well documented in the literature [7]. Properties such as stiffness and compliance directly affect the interaction between the occlusion device and LAA wall, influencing procedural outcomes. The LAA exhibits a distinctive nonlinear stress–strain response, typically represented by a J-shaped curve [7,56]. This curve, characteristic of biological soft tissues, enables the tissue to remain compliant under low loads and progressively stiffen under increasing strain—an essential feature for energy absorption and structural integrity. This behavior is attributed to the native fiber-reinforced architecture of the extracellular matrix, which governs the tissue’s mechanical adaptation [1,12]. Therefore, for a synthetic model to accurately replicate LAA mechanics, the selected material must reproduce not only the strain capacity and strength but also the overall geometry of the stress–strain curve.

The results shown in Figure 8 indicate a clear divergence between the mechanical behavior of TPU 85A and TPE 60A and LAA biological tissue. TPU 85A exhibited a significantly higher response to tensile stress, particularly in samples with 25% infill, reaching approximately 226 kPa at 60% strain. This represents a significantly higher stress value, approximately 46.9% greater than that of LAA tissue, which does not exceed 120 kPa. TPU 85A’s mechanical behavior suggests high stiffness, which makes it inappropriate for applications that intend to replicate the flexibility of biological tissues such as LAA tissues.

At a 10% infill, the TPU 85A material still exhibited substantially higher tensile stress than LAA tissue. Conversely, TPE 60A exhibited a more flexible behavior at both infill density percentages, with a maximum tensile stress of 80 kPa (10% infill) and 106 kPa (25% infill) at 60% strain. However, despite presenting similar values in terms of strain, TPE still exhibited higher tensile stress than LAA biological tissue, especially in 25% infill density percentage samples. Even in 10% infill density samples, despite having the stress–strain curve which most resembles that of LAA tissue, it still displayed a considered difference in tensile stress, suggesting that the material is suitable for replicating the mechanical behavior of the LAA but not suitable when considering the values of its mechanical properties.

The results for TPE 70A for both infill variations, for the same number of shells, 0S + 10% and 0S + 25%, indicate that this material has an intermediary mechanical behavior compared to the other tested specimens. TPE 70A with 0S + 25% demonstrated a good replication of the profile of the stress–strain curve of LAA tissue, particularly until 40% deformation. In this strain range, TPE 70A with 0S + 25% exhibited higher tensile stress values than the LAA, reflecting greater stiffness relative to the target tissue. Despite this, the stress–strain curve of TPE 70A with 0S + 25% still shows a reasonable correlation with the characteristic stress–strain behavior of the LAA, particularly in terms of its overall shape and trend within this strain range. However, it is possible to observe that, from 40% strain onwards, there is an alteration in the mechanical behavior, indicating a different tendency towards higher strain values while exhibiting lower tensile stress.

On the other hand, regarding the overall strain range, TPE 70A samples with 0S + 10% presented lower tensile stress and stiffness values. Regardless of both TPE 70A infill variations exhibiting similarities in their stress–strain curves up to approximately 25% strain, beyond this point, samples with 0S + 10% exhibited an increase in stress–strain values, although with a reduced rate of progression. It is important to mention that, at 60% strain, both specimens of TPE 70A (0S + 10% and 0S + 25%) exhibited maximum stress values lower than those of LAA tissue, suggesting that the material is mechanically less resistant than the biological tissue.

Among all the parameters studied, the results highlight the adjustment capacity of the selected materials, given that variations in infill density resulted in distinct mechanical responses. Both TPU 85A and TPE 60A exhibit significantly steeper curves, indicating that these materials with the tested printing parameters are not suitable for replicating the behavior of LAA. Regarding TPE 70A, despite its divergence from the final part of the LAA’s curve, particularly for the 0S + 25% printing parameters, its stress–strain behavior until 40% strain was similar, and its maximum tensile stress value was the closest, with a difference of 13.2%. The major differences that were observed concerning the nonlinear response at higher strain levels highlight the need for small changes in manufacturing conditions. These adjustments can improve the alignment with the behavior of biological tissue, especially at higher strain values.

The stress–strain curve considered for comparison (Figure 2) is the stress–strain curve of a healthy LAA. However, according to the literature, the LAA is more prone to blood clot generation in patients predisposed to atrial fibrillation episodes. The tissues of these LAAs typically exhibit stiffer and less linear mechanical behaviors [57]. Among the tested materials, TPE 70A’s characteristics most closely resemble these characteristics. As our idea was to create three-dimensional models to allow physicians to plan and practice LAA occlusion procedures, selecting a higher infill density percentage is advantageous. This choice ensures that the occlusion device can be more effectively anchored, mimicking the mechanical properties required for clinical success. Therefore, the material that best replicates the mechanical behavior of LAA tissue is TPE 70A printed with zero shells, 25% infill, and a linear 45°/−45° infill pattern.

## 4. Conclusions

This research aimed to identify synthetic materials and printing parameters to mimic the mechanical behavior of LAA biological tissue. Among the selected materials, TPU and TPE, tensile test results indicated that the most suitable materials for this study’s objective were the ones with lower hardness values, TPU 85A, TPE 70A, and TPE 60A, as they exhibited deformation values closer to those of the LAA. However, the results were not ideal, making it necessary to study several printing parameters.

Shell number (zero, one, and two) and infill density (0%, 10%, 25%, 50%, and 100%) were analyzed. It was concluded that, regardless of the printing parameters, TPU 85A, due to its higher stress–strain response, demonstrates characteristics that make it unsuitable for applications requiring greater flexibility, as typically observed in biological tissues. On the other hand, TPE 60A is a more flexible material and exhibits lower tensile values than LAA biological tissue. For samples with zero shells and 10% infill density, TPE 60A can accurately replicate the mechanical behavior of LAA up to 30% strain. Nonetheless, the stress–strain curves diverge for higher strain values, with TPE 60A showing lower tensile stress values than LAA tissue. Regarding TPE 70A, specimens printed with 0S + 10% exhibited some initial similarities to the mechanical behavior of LAA tissue, particularly at strain values lower than 26% when comparing the stress–strain curves of 0S + 10% and LAA biological tissue. However, in addition to exhibiting higher maximum stress values than those of the biological tissue throughout the entire analyzed range, its stress–strain curve reveals a mechanical behavior which was less representative of LAA tissue at higher strain values. Additionally, the samples produced with these printing parameters show a tendency for lower stiffness compared to the 0S + 25% ones, which can reduce their applicability for models that aim to simulate pathological conditions of the LAA (where the tissue behaves more stiffly and in a less linear manner). Furthermore, the anchoring process of occlusion devices can be compromised in models based on these printing parameters due to low structural density.

TPE 70A with the 0S + 25% configuration was identified as the most suitable option to replicate the mechanical characteristics of LAA tissue under pathological conditions, such as those observed in patients predisposed to clot formation during episodes of atrial fibrillation. Although the material displays higher tensile stress values than LAA tissue, its stress–strain behavior is the closest compared to the other evaluated materials, especially at strain values lower than 40%. Moreover, a 25% infill enables more effective anchoring of the occlusion device, allowing for more reliability in LAA occlusion procedural planning. Therefore, TPE 70A with 0S + 25% infill density was selected for developing LAA tridimensional models due to its capacity to attend to the mechanical behavior characteristics of LAA tissue while simultaneously facilitating the clinical practice of occlusion procedures.

This study marks a significant advancement in identifying materials suitable for mimicking the LAA. In fact, although some studies focus on the LAA, most are numerical, providing little to no insight into the mechanical properties of used materials.

## 5. Future Research and Limitations of the Study

The main results of this study are promising since the stress–strain curves of the synthetic materials closely resemble the mechanical response of LAA biological tissue. However, for accurate replication of this behavior, the study highlights the necessity of approaching novel infill patterns, maintaining selection of materials with zero shells and low infill density values. By isolating the infill pattern as a variable of interest, it is possible to better understand its impact on these materials’ mechanical properties and overall effectiveness. This method could result in improved material designs that closely resemble the properties of LAA tissue and enhance progress in creating biomimetic structures for medical purposes.

As a limitation of this research, the lack of studies regarding the mechanical characterization of LAA biological tissues is underlined. The comparison between biological tissue and the synthetic materials was performed based only on a single LAA stress–strain curve. Despite the expected range of values of mechanical properties, LAA biological tissue characteristics are individual since they are affected by several factors, such as associated cardiac pathology and physical activity. So, the use of one LAA tensile stress–strain curve led to preliminary results.

## Figures and Tables

**Table 1:** Principal printing parameters for each material.

Material	Extrusion Temperature (°C)	Bed Temperature (°C)	Printing Speed (mm·s^−1^)
TPU 100A	210	40	40
TPU 93A	225	50	40
TPU 90A	220	60	40
TPU 85A	230	60	40
TPE 70A	230	60	40
TPE 60A	220	30	60

**Table 2:** Average and standard deviation values of σ and E of the printed materials using two shells and 100% infill.

Material	σ (MPa)	E (kPa)
TPU 100A	6.8 ± 0.7	409.6 ± 19.6
TPU 93A	5.6 ± 0.6	369.8 ± 59.7
TPU 90A	5.0 ± 0.6	309.9 ± 60.2
TPU 85A	2.6 ± 0.8	113.9 ± 5.8
TPE 70A	1.3 ± 0.2	36.3 ± 1.9
TPE 60A	0.8 ± 0.1	20.6 ± 1.9

**Table 3:** Average and standard deviation values of σ and E of TPU 85A, TPE 70A, and TPE 60A, according to different sets of printing parameters.

Material	Printing Parameters	σ (MPa)	E (kPa)
TPU 85A	2S + 100%	2590 ± 800	113.9 ± 5.8
	2S + 50%	1520 ± 74	53.5 ± 8.7
	2S + 25%	1040 ± 14	28.4 ± 1.2
	2S + 0%	390 ± 40	10.9 ± 1.2
	1S + 100%	2022 ± 300	73.6 ± 5.2
	1S + 50%	1080 ± 124	29.4 ± 3.4
	1S + 25%	730 ± 90	23.1 ± 1.3
	1S + 0%	330 ± 60	9.6 ± 0.7
TPE 70A	2S + 100%	1331 ± 154	36.3 ± 1.9
	2S + 50%	736 ± 95	18.7 ± 2.2
	2S + 25%	650 ± 49	15.3 ± 1.5
	2S + 0%	265 ± 5	6.2 ± 0.5
	1S + 100%	1226 ± 48	33.5 ± 1.1
	1S + 50%	597 ± 4	14.5 ± 1
	1S + 25%	397 ± 7	9.1 ± 0.1
	1S + 0%	220 ± 3	6.1 ± 0.7
TPE 60A	2S + 100%	825 ± 30	20.6 ± 1.9
	2S + 50%	412 ± 30	11.8 ± 0.9
	2S + 25%	362 ± 18	8.7 ± 0.8
	2S + 0%	222 ± 40	4.0 ± 0.3
	1S + 100%	469 ± 30	14.1 ± 2.3
	1S + 50%	333 ± 20	8.1 ± 0.7
	1S + 25%	253 ± 0.021	4.9 ± 0.2
	1S + 0%	125 ± 0.05	3.1 ± 0.1

**Table 4:** Average and standard deviation values of σ and E of TPU 85A, TPE 70A, and TPE 60A for different printing parameter combinations.

Material	Printing Parameters	σ (kPa)	E (kPa)
TPU 85A	0S + 10%	114 ± 21	2.7 ± 0.3
	0S + 25%	226 ± 25	1.1 ± 0.2
TPE 70A	0S + 10%	80 ± 3	1.0 ± 0.1
	0S + 25%	106 ± 11	1.0 ± 0.1
TPE 60A	0S + 10%	40 ± 2	0.8 ± 0.1
	0S + 25%	72 ± 5	0.3 ± 0.1

## Data Availability

The original contributions presented in this study are included in the article. Further inquiries can be directed to the corresponding authors.
